# Comparative Analysis of Adipokines and Growth Factors in Preterm and Term Breast Milk and Their Role in Infant Growth

**DOI:** 10.1007/s13668-026-00758-0

**Published:** 2026-04-22

**Authors:** Tugce Tekin Guler, Mehmet Fisunoglu

**Affiliations:** 1 Department of Nutrition and Dietetics, Faculty of Health Sciences, Hacettepe University, Altindag, Ankara Türkiye; 2 Department of Nutrition and Dietetics, Faculty of Health Sciences, Eskisehir Osmangazi University, Odunpazari, Eskisehir Türkiye

**Keywords:** Breast milk, Human milk, Preterm, Adipokines, Growth factors

## Abstract

**Purpose of Review:**

Since breast milk provides essential nutrients and bioactive compounds, such as adipokines and growth factors, which are indispensable for neonatal growth and metabolic regulation, this review seeks to elucidate the differences in these bioactive components between preterm and term breast milk and to evaluate their potential influence on neonatal development.

**Recent Findings:**

Adipokines such as leptin, adiponectin, resistin, ghrelin, and visfatin, alongside growth factors including epidermal growth factor, insulin-like growth factor, and brain-derived neurotrophic factor, exhibit variable concentrations in preterm versus term milk. Preterm milk generally contains higher levels, potentially reflecting an adaptive response to support accelerated growth. However, findings are inconsistent across studies, likely due to differences in study design, timing of milk collection, analytical methods, and variability in maternal characteristics.

**Summary:**

Variations in bioactive component profiles between preterm and term milk suggest compensatory mechanisms in preterm lactation. Further longitudinal studies are warranted to clarify these relationships and to elucidate the long-term effects of breast milk adipokines and growth factors on preterm infant growth and metabolic programming.

## Introduction

Breast milk is widely recognized as the gold standard for infant nutrition, providing not only essential macronutrients and micronutrients but also a complex array of bioactive factors that contribute to immune protection, organ maturation, and long-term metabolic programming [1, 2]. Among these bioactive molecules, adipokines and growth factors have emerged as pivotal regulators of neonatal metabolic processes and developmental trajectories [3, 4]. Their dynamic presence and variation in breast milk underscore a finely tuned biological system tailored to the infant’s needs [5].

In terms of its basic composition, breast milk is made up of approximately 87% water, along with macronutrients such as lipids (3–5%), carbohydrates (6.9–7.2%), proteins (0.8–1%), and small amounts of vitamins and minerals (around 0.2%) [6]. Notably, term and preterm breast milk differ significantly, with preterm colostrum containing up to 35% more true protein during the early postpartum days, although this difference tends to diminish after the first week [7]. Preterm milk may also provide slightly higher fat and energy levels in the early postpartum period; however, term milk can become more energy-dense during weeks 3–8 of lactation [8, 9]. Despite these early differences, the concentrations of protein and lactose in both types of milk generally converge over time, becoming largely similar [7].

The composition of breast milk is distinctive and dynamic, varying not only within a single feeding but also throughout lactation [10]. Importantly, breast milk composition is shaped by a range of maternal and infant-related factors—including the infant’s gestational age, lactation stage, maternal parity, health status, and diet—as well as by environmental conditions and transient physiological or metabolic states [10, 11]. The interplay between maternal and infant physiology ensures that the bioactive composition of milk is continually adapted to meet the evolving developmental needs of the infant [5, 12]. In addition to nutrients, breast milk contains a wide spectrum of bioactive molecules that support immune defense, organ maturation, and long-term metabolic programming [5, 12]. Among these, adipokines and growth factors represent key regulatory elements [3, 4]. These components not only reflect the mother’s physiological and metabolic status but also actively participate in shaping the infant’s development trajectory [13–15].

Preterm birth remains a significant global health challenge, with an estimated 13.4 million infants born before 37 weeks of gestation in 2020 alone [16]. Infants born at a gestational age of fewer than 37 weeks are defined as preterm infants. Preterm infants are categorized as extremely preterm if born at less than 28 weeks, very preterm between 28 and 32 weeks, and moderate to late preterm if born between 32 and 37 weeks [17]. Breast milk is undoubtedly the optimal source of nutrition for preterm infants, just as it is for term infants [18, 19]. In addition to the macro and micronutrient requirements of preterm infants, adipokines and growth factors in breast milk play a significant role in ensuring adequate growth and development [20, 21]. Although breast milk is the optimal source of nutrition for both term and preterm infants, it often fails to meet the heightened nutritional demands of very low birth weight preterm infants when unfortified [22]. To meet the high nutritional demands of very preterm infants and reduce the risk of extrauterine growth restriction and its adverse neurodevelopmental consequences, human milk must be fortified, as its native composition alone is insufficient when provided in typical feeding volumes [23].

Preterm infants are susceptible to a wide range of life-threatening morbidities, including necrotizing enterocolitis, sepsis, bronchopulmonary dysplasia, and retinopathy of prematurity, which reflect their physiological vulnerability during the neonatal period [24]. In addition, due to their immature organ systems and elevated nutritional demands, they are at increased risk for extrauterine growth restriction and long-term developmental impairments [25]. In this context, the bioactive components of breast milk, particularly adipokines and growth factors, become even more critical in supporting catch-up growth and promoting healthy development [5, 12]. These molecules function not merely as passive nutritional elements but as active endocrine and paracrine signals that help regulate appetite, energy balance, tissue growth, and metabolic adaptation in the neonatal period [3, 13]. Leptin in breast milk may play a key role in shaping neonatal appetite regulation and energy balance, acting through endocrine pathways that influence hypothalamic development and metabolic programming [2, 3, 13]. Similarly, insulin-like growth factor (IGF) −1 and epidermal growth factor (EGF) are essential for intestinal growth and mucosal integrity, thereby supporting efficient nutrient absorption and growth in preterm infants [5, 26]. Adipokines such as adiponectin and ghrelin may also mediate glucose metabolism and lipid mobilization, processes that are vital during the rapid postnatal growth phase [27]. Adipokines such as leptin, adiponectin, resistin, ghrelin, obestatin, nesfatin, apelin, and visfatin play a crucial role in infants’ metabolism and growth [2, 28]. Additionally, growth factors such as IGF, EGF, brain-derived neurotrophic factor (BDNF), and vascular endothelial growth factor (VEGF) are also important in the growth and development of the infant [5, 26]. Thus, these bioactive components do not only reflect maternal physiological states but also act as developmental regulators that can shape the trajectory of catch-up growth in preterm infants [5, 13, 23].

This review will explicitly explore the variations in the concentrations of bioactive compounds in breast milk, such as adipokines and growth factors, in particular between milk produced by mothers of preterm versus term infants. It will discuss how these compounds potentially modulate growth patterns, including weight gain, length gain, and z-scores for these parameters. Additionally, the review will discuss the relationships between these bioactive components and factors that influence breast milk composition, such as maternal BMI, maternal health status, and maternal diet. In this context, the objective is to summarize existing research on the differences in adipokine and growth factor levels in the breast milk produced by mothers of preterm versus term infants and to evaluate their influence on infant growth.

## Methods of Literature Search

Data obtained from human studies were evaluated to assess the changes in adipokine and growth factor levels in breast milk produced by mothers of preterm infants and their impact on infant growth. The data were collected through searches performed across various databases, including PubMed, ScienceDirect, Web of Science, and Scopus, with access provided by Hacettepe University Library, from inception through July 2025. The following keywords were relevant to the study: breast milk, human milk, preterm infants, premature, bioactive components, immunological factors, hormones, adipokines, and growth factors. This review includes only studies that focused on analyzing preterm breast milk or on comparing preterm and term breast milk. Studies involving donor milk were excluded to ensure consistency in maternal-origin milk composition. Donor milk is typically pasteurized and pooled from multiple sources, which may alter the concentration and bioactivity of components such as adipokines and growth factors. Including such studies could confound the interpretation of findings related to directly expressed maternal preterm milk. Additionally, studies not published in English were excluded. As a result of this review, 27 studies were selected, and the main results on adipokines and growth factors presented in Table 1, and the results related to infant growth in relation to these components shown in Table 2.

**Table 1:** Studies exploring the adipokines and growth factors in breast milk from preterm deliveries

Author, Year	Preterm/ Term (*n*; gestastional age)	Timing of Collection	Analysis	Adipokines & Growth Factor	Main Results
C. Balcells-Esponera et al. (2024)[29]	Preterm (*n* = 100; 28.8 ± 2.3 weeks)	Day 28	Leptin, adiponectin: ELISA	Leptin: 531.5 ± 419.8 pg/mL Adiponectin: 25.4 ± 8.0 ng/mL	Mothers with gestational diabetes had higher levels of adiponectin (30.7 ± 6.5 vs. 24.8 ± 8 ng/mL, *p* = 0.044). Additionally, leptin concentrations showed a significant association with pre-pregnancy BMI (*p* < 0.0001).
M. C. Collado et al. (2015)[27]	Preterm (*n* = 20; 29 weeks) Term (*n* = 20; 39.2 weeks)	Colostrum (1–6 days) Transitional (7–15 days) Mature milk (> 15 day)	EGF, IGFBP-1, IGFBP-2: protein array	Neurotrophic factors and other growth factors: Higher in preterm than term	Mature milk samples produced by mothers of preterm infants displayed significantly higher expression of neurotrophic factors than those produced by mothers of term infants. The term group had higher concentrations of IGFBP-1, whereas the preterm group showed increased levels of EGF and IGFBP-2.
C. Castellote et al. (2011)[30]	Very preterm (*n* = 10; 26.7 ± 0.37 weeks) Preterm (*n* = 10; 34.0 ± 0.62 weeks) Term (*n* = 22; 40.6 ± 0.21 weeks)	Colostrum (3 ± 1 days) Transitional (10 ± 2 days) Mature milk (30 ± 2 days)	EGF, TGFβ1, TGFβ2: ELISA	EGF: higher in preterm and very preterm (transitional) TGF-β1: highest in preterm (colostrum) TGF-β2: higher in preterm (colostrum)	Colostrum produced by mothers of preterm infants was notably abundant in most of the studied factors, although significantly higher concentrations compared with colostrum produced by mothers of term infants were only observed for TGF-β1 and TGF-β2 (*p* < 0.05).
R. Mehta et al. (2011)[31]	Preterm (*n* = 15; 28 ± 2.2 weeks) Term (*n* = 5; 38.8 ± 0.8 weeks)	Transitional hind milk (6–8 and 13–15 days) Mature hind milk (20–22 and 27–29 days)	Leptin, adiponectin: ELISA	NA	Adiponectin concentrations were higher in breast milk produced by mothers of preterm infants than in breast milk produced by mothers of term infants, whereas leptin levels were higher in breast milk produced by mothers of term infants than in that produced by mothers of preterm infants (*p* < 0.05).
B. Kociszewska-Najman et al. (2020)[32]	Preterm (*n* = 48; <37 weeks) Term (*n* = 251; ≥37 weeks)	Day 5	TGF-β2: ELISA	TGF-β2-Preterm: 4648 ± 772 ng/mL TGF-β2-Term: 3899 ± 533 ng/mL	TGF-β2 concentrations in colostrum were higher in breast milk produced by mothers of preterm infants than in that produced by mothers of term infants, although the difference was not statistically significant (*p* = 0.1244).
S. Trend et al. (2016)[33]	Extremely preterm (*n* = 15; 26.1 ± 1.2 weeks) Very preterm (*n* = 15; 30.2 ± 1.3 weeks) Moderately preterm (*n* = 15; 34.4 ± 1.3 weeks) Term (*n* = 15; 39.4 ± 0.77 weeks)	Colostrum (2–5 days), transitional (8–12 days) and mature milk (26–30 days)	TGF-β2: ELISA	TGF-β2: 12.56 ng/mL	The concentration of TGF-β2 in colostrum was significantly higher in mothers of extremely preterm infants than in mothers of term infants.
M. D. Caba-Flores et al. (2023)[34]	Extremely and very preterm (*n* = 10; 28.94 ± 1.5 weeks) Moderately preterm (*n* = 21; 34.1 ± 1.48 weeks)	21–30 days	Ghrelin, leptin: Luminex multiplexing assay	NA	There were no significant differences in ghrelin and leptin levels related to preterm status, gestational status, infant sex at birth, or maternal BMI.
D. Ramiro-Cortijo et al. (2023)[35]	Preterm (*n* = 18; 28.7 weeks) Term (*n* = 30; 38.9 weeks)	7, 14, and 28 days	Ghrelin, resistin, leptin: A Human U-Plex Ultra-Sensitive Meso Scale Discovery Kit	NA	Compared to term breast milk, preterm breast milk contained significantly higher leptin and resistin levels, while ghrelin levels were significantly lower.
B. Kocaadam et al. (2019)[36]	Preterm (*n* = 31; 35.3 weeks) Term (*n* = 34; 38.7 weeks)	15–30 days	Adiponectin, leptin: ELISA	Preterm Adiponectin: 24.6 ng/ml Leptin: 2.0 ng/ml Term Adiponectin: 22.9 ng/ml Leptin: 0.0 ng/ml	A higher median level of adipokines was detected in breast milk from mothers of preterm infants (*p* > 0.05).
M. Slupecka-Ziemilska et al. (2017)[37]	Preterm (*n* = 20; 33.8 ± 2.3 weeks) Term (*n* = 20; 39 ± 1 weeks)	Day 3	Ghrelin, obestatin: RIA kit	NA	No significant differences were observed in ghrelin levels between the preterm and term groups, while obestatin was found to be significantly lower in the preterm group.
E. Eilers et al. (2011)[38]	Preterm (*n* = 37; 32 + 1± 3 + 5 weeks) Term (*n* = 40; 39 + 4± 1 + 4 weeks)	3 and 28 days	Leptin: RIA kit	Leptin-Preterm day 3: 0.70 ± 0.79 ng/mL day 28: 0.50 ± 0.40 ng/mL Leptin-Term day 3: 0.65 ± 0.67 ng/mL day 28: 0.50 ± 0.50 ng/mL	There was no difference in leptin levels between preterm and term human milk. On days 3 and 28, leptin levels were positively correlated with maternal BMI but showed no correlation with the fat content of the milk.
A. Oslislo et al. (2007)[39]	Preterm AGA (*n* = 12; 22–31 weeks) Preterm SGA (*n* = 8; 22–31 weeks) Preterm AGA (*n* = 13; 32–36 weeks) Preterm SGA (*n* = 15;32–36 weeks) Full term (*n* = 33)	Colostrum (1–5 days)	EGF: ELISA	Preterm AGA (22–31 weeks): 159.24 ± 51.05 ng/mL Preterm SGA (22–31 weeks): 80.69 ± 33.52 ng/mL Preterm AGA (32–36 weeks): 102.03 ± 55.43 ng/mL Preterm SGA (32–36 weeks): 128.57 ± 49.29 ng/mL Full term: 95.75 ± 48.58 ng/mL	Colostrum from mothers who delivered premature AGA infants at less than 32 weeks gestation showed significantly higher concentrations of EGF compared to mothers who delivered premature SGA infants at the same gestational age.
J. A. Kierson et al. (2006)[40]	Preterm (*n* = 10; 30.4 weeks) Term (*n* = 10; 37.7 weeks)	7–21 days	Ghrelin: RIA	Ghrelin Preterm: 2500 pg/ml Term: 1575 pg/ml	Ghrelin levels did not differ significantly between term and preterm milk samples. Furthermore, a direct correlation was identified between the ghrelin concentration in whole milk and the estimated milk fat content (*r* = 0.84, *p* < 0.001).
J. Bielicki et al. (2004)[41]	Preterm (*n* = 9; 32.11 ± 1.16 weeks) Term (*n* = 24; 39.42 ± 0.22 weeks)	2–3 days, 4–5 days and 6 weeks	Leptin: RIA kit	Preterm (2–3 days): 0.63 ± 0.18 ng/ml Term (2–3 days): 1.34 ± 0.14 ng/ml	Leptin levels were significantly higher in the term group compared to the preterm group on days 2–3 (*p* < 0.01) and days 4–5 (*p* < 0.05).
B. Dvorak et al. (2003)[42]	Extremely preterm (*n* = 16; 23–27 weeks) Preterm (*n* = 16; 32–36 weeks) Term (*n* = 15; 38–42 weeks)	1, 2, and 4 weeks	EGF, TGF-𝑎: RIA	EGF: 40–560 µg/L TGF-𝑎: 0.2–0.6 µg/L	EGF levels were significantly higher in the extremely preterm group (23–27 weeks) compared to both the preterm and full-term groups over the first month of lactation. A comparable pattern was seen with TGF-α, although TGF-α levels were lower than EGF. Significantly higher TGF-α concentrations were observed in the extremely preterm group relative to the preterm or full-term groups. Additionally, TGF-α concentrations in the extremely preterm and preterm groups showed a significant decline between days 7 and 28.
N. Mól et al. (2018)[43]	Very low birth weight preterm (*n* = 53; 30 ± 2.03 weeks) Term (*n* = 19; 39 ± 1.32 weeks)	1, and 5 weeks	Visfatin: ELISA	NA	Visfatin was significantly higher in preterm group during 5th week of life.
M. Groer et al. (2016)[44]	Very-low-birth-weight (*n* = 76; 28.35 ± 2.39 weeks)	1, 2, 3, 4, 5, and 6 weeks	EGF: ELISA	NA	The level of EGF remained elevated throughout the entire data collection period.
B. L. Frost et al. (2014)[45]	Preterm (*n* = 100; <32 weeks)	0–48 h, 6–7 days, and 30–31 days	TGF-β1, TGF-β2: ELISA	NA	Postnatally, TGF-β levels decreased, with the highest concentrations found in colostrum (*p* < 0.01). TGF-β2 concentrations were consistently higher than TGF-β1 at all time points (*p* < 0.01).
T. Ozgurtas et al. (2010)[46]	Preterm (*n* = 29; <37 weeks) Term (*n* = 29; >38 weeks)	3, 7, and 28 days	VEGF, FGF, PDGF: ELISA IGF-1: RIA kit	Preterm (3, 7, 28 d respectively) VEGF: 881, 641, 430 ng/mL FGF: 0.33, 0.33, 0.46 pg/mL IGF-1: 2.31, 3.58, 4.19 ng/mL PDGF: 0.0, 0.0, 0.0 pg/mL Term (3, 7, 28 d respectively) VEGF: 782, 420, 315 ng/mL FGF: 0.0, 0.0, 0.0 pg/mL IGF-1: 0.19, 0.12, 0.31 ng/mL PDGF: 19.6, 0.55, 15.2 pg/mL	The concentrations of VEGF, IGF-1, and FGF were significantly higher in the breast milk of mothers who delivered preterm compared to mothers who delivered at term, while PDGF levels were lower, but the difference was not statistically significant. Longitudinally, there was no statistical difference in FGF concentration from human milk samples of preterm and term groups but, within preterm group concentrations of VEGF, IGF-1 and PDGF significantly differ during postpartum days 3–7–28, whereas in term groups, only concentrations of VEGF significantly differ with time.
A. Loui et al. (2012)[47]	Preterm (*n* = 50; 33.8 weeks) Term (*n* = 49; 39.7 weeks)	3, and 28 days	VEGF: ELISA	Day 3 VEGF-preterm: 37.1 ng/mL VEGF-term: 53.9 ng/mL	High concentrations of VEGF were present in early lactation (day 3) milk from both preterm and term mothers, with lower levels in mature milk (day 28). On day 3, the median concentration of VEGF was significantly lower in preterm milk compared to term milk (37.1 ng/mL vs. 53.9 ng/mL, *p* < 0.01). No difference was found in VEGF levels between preterm and term milk on day 28.
C. Binder et al. (2023)[48]	Preterm (*n* = 136; 29 + 3 weeks)	NA	Adiponectin, leptin: ELISA	Adiponectin: 18.9 ng/mL Leptin: 0.108 ng/mL	A higher protein intake showed a positive correlation with adiponectin (*p* < 0.001) and leptin (*p* = 0.035) levels.
L. Galante et al. (2021)[49]	Moderate-late preterm (*n* = 169; 32 + 0 to 35 + 6 weeks)	5, 10 days and 4 months	Leptin, adiponectin, IGF-1: ELISA	NA	Gestational diabetes was associated with lower adiponectin. Higher adiponectin and lower leptin were associated with higher socioeconomic status, higher maternal education and ability to fully breastfeed at discharge from hospital. Higher leptin was associated with high perceived stress during hospital admission. IGF-1 displayed sex-specific patterns in association with maternal social deprivation.
K. Nyárády et al. (2020)[50]	M. W. Elmlinger et al., 2007	2–4 days	BDNF: ELISA	BDNF Preterm: 0.03 ng/ml Vaginal birth at term: 0.97 ng/ml Caesaarean section at term: 0.24 ng/ml	Preterm birth was associated with a notable reduction in BDNF levels in breast milk.
M. W. Elmlinger et al. (2007)[51]	Preterm (*n* = 30; <31 weeks) Term (*n* = 19; >37 weeks)	7, and 21 days	IGF-1, IGF-2, IGFBP-2, IGFBP-3: RIA	IGF-1 Preterm: 2.8 ± 0.2 ng/ml Term: 2.3 ± 0.1 ng/ml	No significant differences were found between preterm and term milk in the concentrations of IGF-1, IGF-2, IGFBP-2, and IGFBP-3. However, IGF-1 concentrations tended to be higher in preterm milk. Additionally, a strong correlation (*r* = 0.669) was observed between the concentrations of IGF-1 and IGF-2, and these IGF parameters were associated with the total protein content in preterm milk.
H. Itoh et al. (2002)[52]	Preterm (*n* = 10; 33.3 ± 2.4 weeks) Term (*n* = 10; 37.8 ± 0.92 weeks)	Colostrum (1–7 days), transitional milk (8–14 days), and mature milk (30–35 days)	HGF: ELISA	NA	In term deliveries, HGF concentrations exhibited a significant negative correlation with the progression of lactation. In contrast, HGF concentrations from preterm deliveries remained elevated even after extended lactation periods. Preterm deliveries showed significantly higher HGF levels in colostrum, transitional milk, and mature milk compared to term deliveries.

Abbreviations: *AGA* appropriate for gestational age, *BDNF* brain-derived neurotrophic factor, *BMI* body mass index, *EGF* epidermal growth factor, *ELISA* enzyme-linked immunosorbent assay, *FGF* fibroblast growth factor, *HGF* hepatocyte growth factor, *IGF* insulin-like growth factor, *IGFBP* insulin-like growth factor binding protein, *NA* not available, *PDGF* platelet-derived growth factor, *RIA* radioimmunoassay, *SGA* small for gestational age, *TGF* transforming growth factor, *VEGF* vascular endothelial growth factor

Since the levels of adipokines and growth factors in breast milk are given in graphic form in some articles, exact values could not be obtained

**Table 2:** Studies examining the association between adipokines and growth factors in breast milk from preterm deliveries and infant growth

Author, Year	Preterm/ Term (*n*; gestastional age)	Timing of Collection	Analysis	Infant Growth/ Infant Anthropometry
C. Balcells-Esponera et al. (2024)[29]	Preterm (*n* = 100; 28.8 ± 2.3 weeks)	Day 28	Leptin, adiponectin: ELISA	Milk adiponectin was associated with a greater decrease in length z-scores from birth to 28 days (Pearson’s *r* = −0.225, *p* = 0.032) and to discharge (Pearson’s *r* = −0.290, *p* = 0.003).
C. Castellote et al. (2011)[30]	Very preterm (*n* = 10; 26.7 ± 0.37 weeks) Preterm (*n* = 10; 34.0 ± 0.62 weeks) Term (*n* = 22; 40.6 ± 0.21 weeks)	Colostrum (3 ± 1 days) Transitional (10 ± 2 days) Mature milk (30 ± 2 days)	EGF, TGFβ1, TGFβ2: ELISA	Transitional milk from all groups showed a negative correlation between EGF levels and birth weight (*r* = −0.57; *p* = 0.001).
D. Ramiro-Cortijo et al. (2023)[35]	Preterm (*n* = 18; 28.7 weeks) Term (*n* = 30; 38.9 weeks)	7, 14, and 28 days	Ghrelin, resistin, leptin: A Human U-Plex Ultra-Sensitive Meso Scale Discovery Kit	In preterm infants, weight and length gain were positively correlated with ghrelin levels in breast milk, with an increase in ghrelin on day 14 being associated with greater growth.
L. Galante et al. (2021)[49]	Moderate-late preterm (*n* = 169; 32 + 0 to 35 + 6 weeks)	5, 10 days and 4 months	Leptin, adiponectin, IGF-1: ELISA	On day 5 post-birth, higher IGF-1 levels in breast milk were associated with increased fat-free mass at 4 months’ corrected age. At 4 months, IGF-1 concentrations in breast milk were positively correlated with both fat mass and fat-free mass in boys, but not in girls. Leptin levels in breast milk on day 5 were positively associated with fat mass at discharge but negatively correlated with fat mass at 4 months’ corrected age.
K. E. Joung et al. (2021)[53]	Preterm (*n* = 50; 29.4 weeks)	7, 14, 21, and 28 days	Leptin, adiponectin, insulin: ELISA	Higher leptin intake was associated with greater weight gain (2.17 g/kg/day [95% CI, 1.31, 3.02]) and weight z-score at 36 weeks’ postmenstrual age (0.30 [0.08, 0.53] higher z-score per tertile). Higher adiponectin intake was associated with greater length z-score (0.41 [0.13, 0.69]), however, this association was nullified after adjustment of protein and calorie intake. Higher adiponectin was associated with smaller head circumference z-score (−0.36 [−0.64, −0.07]).
B. Kocaadam et al. (2019)[36]	Preterm (*n* = 31; 35.3 weeks) Term (*n* = 34; 38.7 weeks)	15–30 days	Adiponectin, leptin: ELISA	In preterm infants, breast milk adiponectin levels were negatively correlated with length gain and positively correlated with BMI increase during the second and third months. Additionally, leptin levels were negatively correlated with head circumference at birth in preterm infants (*p* < 0.05).
J. Bielicki et al. (2004)[41]	Preterm (*n* = 9; 32.11 ± 1.16 weeks) Term (*n* = 24; 39.42 ± 0.22 weeks)	2–3 days, 4–5 days and 6 weeks	Leptin: RIA kit	A moderate correlation was observed between leptin levels and maternal as well as infant weight or BMI.
B. L. Frost et al. (2014)[45]	Preterm (*n* = 100; <32 weeks)	0–48 h, 6–7 days, and 30–31 days	TGF-β1, TGF-β2: ELISA	TGF-β levels in colostrum showed a negative correlation with birth weight (*p* < 0.01) and gestational age (*p* < 0.05).

Abbreviations: *BMI* body mass index, *EGF* epidermal growth factor, *ELISA*, enzyme-linked immunosorbent assay, *IGF* insulin-like growth factor;* RIA* radioimmunoassay, *TGF* transforming growth factor

### Adipokines

Adipokines in preterm breast milk, such as leptin, adiponectin, and ghrelin, are more than passive components as they actively contribute to energy regulation, appetite control, and overall growth processes in neonates [12]. Their concentrations are influenced by maternal characteristics including pre-pregnancy BMI, metabolic conditions, and stress levels, which may indirectly shape the infant’s early developmental environment [3, 54]. This underscores the dynamic and responsive nature of breast milk composition and emphasizes the role of adipokines as critical contributors to early-life metabolic programming [3, 12].

## Leptin

Leptin in breast milk acts as a neurotrophic factor by improving neuronal connectivity and influencing eating behaviors [55]. It also enhances leptin and insulin sensitivity, which may contribute to better control of body weight later in life [55]. Leptin levels in the breast milk produced by mothers of preterm infants range from 0.5 to 2.0 ng/mL [36, 38]. Leptin in preterm breast milk positively correlated with pre-pregnancy body mass index (BMI) and maternal weight [29, 38]. Some studies reported that leptin levels are higher in milk produced by mothers of term infants than in milk produced by mothers of preterm infants [31, 41], while other studies reported the opposite, with higher leptin levels in milk produced by mothers of preterm infants than in milk produced by mothers of term infants [35, 36]. Moreover, other studies found no relationship between leptin levels and preterm status [34, 38]. The discrepancies in leptin levels between breast milk produced by mothers of preterm and term infants may stem from several factors. First, the timing of milk collection during lactation can significantly impact the composition of breast milk, which varies at different stages. Additionally, the small sample sizes in some studies may have limited their power to detect meaningful differences, contributing to the inconsistency in the findings. Furthermore, differences in analytical methods, including assay techniques and sensitivities, could also explain the observed variability in leptin levels.

Studies investigating leptin levels in breast milk and other maternal factors among mothers of preterm infants have shown that leptin concentrations rise in response to increased maternal dietary protein intake and heightened maternal stress [48, 49].

Studies exploring the connection between breast milk leptin in mothers of preterm infants and anthropometric measurements in preterm infants have shown a negative correlation between breast milk leptin levels and both the head circumference at birth and fat mass at a corrected age of 4 months [36, 49]. In addition, the results from two studies revealed that leptin concentration at discharge was positively linked to fat mass, as well as to higher weight gain and weight z-scores at 36 weeks of postmenstrual age [49, 53]. In another investigation, a moderate correlation was observed between breast milk leptin levels and the infant’s weight or body mass index [41]. Although the relationship between leptin in breast milk and preterm status remains inconsistent across studies, findings indicating a correlation between leptin levels and infant fat mass, weight gain, and body composition provide important insights into leptin’s role in infant growth.

## Adiponectin

Adiponectin is implicated in the regulation of both lipid and glucose metabolism, the modulation of food intake, the reduction of energy expenditure, and the control of inflammatory processes [56]. In research conducted on mothers of preterm infants, adiponectin levels in breast milk were observed to range between 18.9 and 25.4 ng/mL, which were significantly higher than leptin levels [29, 48]. Two studies that compared adiponectin levels in the breast milk produced by mothers of term and preterm infants reported higher adiponectin levels in milk produced by mothers of preterm infants [31, 36]. In one study, adiponectin levels in breast milk were found to be elevated in mothers with gestational diabetes and preterm infants compared to other mothers [29]. The elevated adiponectin levels in breast milk produced by mothers of preterm infants may reflect the body’s attempt to compensate for the underdeveloped metabolic and immune systems, promoting a more efficient regulation of energy and fat storage.

A positive correlation between adiponectin levels and maternal dietary protein intake, socioeconomic status, maternal education, and the ability to exclusively breastfeed at discharge has been observed [48, 49].

Studies have shown that adiponectin levels in breast milk are negatively correlated with the infant’s head circumference z-score and length z-score [29, 36, 53] while being positively correlated with the increase in BMI [36]. Given the positive association between higher adiponectin levels in breast milk from mothers of preterm infants and increased infant BMI, it is likely that these elevated levels influence the long-term growth trajectory of preterm infants.

## Ghrelin

Ghrelin significantly impacts food intake, appetite regulation, glucose and lipid metabolism, energy homeostasis, and body weight regulation [15]. In breast milk produced by mothers of preterm infants, ghrelin concentration has been reported to be 2500 pg/mL [40]. When examining ghrelin levels in breast milk produced by mothers of preterm and term infants, one study found significantly lower ghrelin concentrations in the breast milk from mothers of preterm infants compared with those of term infants [35], while three studies reported no significant difference between the groups [34, 37, 40]. In preterm neonates, weight gain and length gain were associated with increased breast milk ghrelin levels at day 14 [35]. According to the majority of studies available, prematurity does not seem to significantly affect ghrelin levels in breast milk.

## Resistin

Resistin plays an antagonistic role in insulin function by impairing glucose homeostasis [57]. Only one study has investigated the resistin levels in the breast milk produced by mothers of preterm infants. Based on the study, resistin levels in breast milk were significantly higher in milk produced by mothers of preterm infants than in milk produced by mothers of term infants and decreased throughout the first month of lactation [35]. The elevated resistin levels in the breast milk produced by mothers of preterm infants may be a compensatory mechanism to mitigate insulin’s effects, potentially helping to prevent hypoglycemia. However, further studies are required to fully understand the physiological significance of these findings.

### Obestatin

Obestatin regulates energy homeostasis and functions as an antagonist to ghrelin [58]. In comparison to the term group, obestatin levels in breast milk produced by mothers of preterm infants were significantly lower [37]. Although studies on obestatin levels in breast milk from mothers of preterm infants are limited, the lower levels observed may represent an adaptive mechanism to support increased nutrient intake, considering obestatin’s appetite-suppressing function in energy regulation.

## Visfatin

Visfatin is an adipokine that plays a key role in regulating insulin resistance and exerts significant effects on both energy metabolism and immune system activity [59]. Visfatin levels in the breast milk collected at the first and fifth weeks showed a progressive increase over four weeks and were significantly higher in milk produced by mothers of preterm infants than in that from mothers of term infants at week 5 of life [43]. The elevated visfatin levels in preterm breast milk may represent a compensatory response aimed at enhancing metabolic support and immune function in neonates with immature physiological systems.

## Growth Factors

Growth factors in breast milk, such as EGF, BDNF, IGF, and VEGF, regulate cell growth, differentiation, and organ development in neonates [2, 5]. These growth factors not only facilitate the maturation of the infant’s gastrointestinal and immune systems but also support neural development and tissue repair [5]. EGF, for instance, has been shown to enhance the integrity of the gut mucosa, which is crucial for preventing necrotizing enterocolitis, a common and serious condition in preterm infants [60]. Additionally, IGF is involved in regulating growth velocity and tissue repair, playing a significant role in the early life growth trajectory of preterm infants [61]. These factors highlight the importance of breast milk as a dynamic source of bioactive compounds that support optimal growth and development in both preterm and term infants [62].

### Epidermal Growth Factor

The role of EGF encompasses promoting cell growth, proliferation, differentiation, intestinal development, and maintenance of intestinal barrier function [63]. EGF is one of the growth factors that is abundantly present in breast milk from mothers of both term and preterm infants [27]. EGF levels in breast milk samples collected weekly from the first to the sixth week in preterm infants were consistently high during the entire data collection period [44]. In similar studies on EGF, EGF concentrations in transitional milk were found to be higher in the preterm and very preterm groups compared to the term group [30], and during the first month of breastfeeding, EGF levels were significantly elevated in the extremely preterm group compared to both the preterm and full-term groups [42]. Moreover, when comparing mothers who delivered premature appropriate for gestational age infants at less than 32 weeks of gestation with those who delivered premature small for gestational age infants at the same gestational age, significantly higher EGF concentrations were found in the colostrum of the former group [39]. The higher EGF levels observed in preterm milk may serve as a compensatory mechanism to support the rapid development of critical tissues, particularly the gut epithelium, in vulnerable preterm neonates.

In the only study examining the relationship between EGF concentration and the anthropometric measurements of preterm infants, a negative correlation was found between EGF concentration in transitional milk and birth weight in both the preterm and very preterm groups [30]. This inverse association may indicate a compensatory increase in EGF levels in response to lower birth weight, aiming to enhance postnatal growth and organ maturation in smaller preterm infants.

### Insulin-Like Growth Factor

IGF-1 and IGF-2, primarily bound to IGF-binding proteins (IGFBP), play a role in intestinal development, as well as in the physiology, growth, and metabolism of cells [64, 65]. Studies showed that while IGF-1 concentration tended to be higher in the breast milk produced by mothers of preterm infants, no significant difference was observed in IGF-2 levels [46, 51]. Moreover, a strong correlation existed between IGF-1 and IGF-2 concentrations, and IGF parameters were found to be associated with protein concentration in breast milk [51]. It was observed that IGFBP-1 and IGFBP-2 were abundant in breast milk produced by mothers of term infants, whereas IGFBP-2 was abundant in breast milk produced by mothers of preterm infants [27]. Furthermore, no statistically significant difference was observed in the levels of IGFBP-2 and IGFBP-3 between breast milk produced by mothers of preterm and term infants [51]. Positive associations were found between IGF-1 levels on day 5 postpartum and fat-free mass at 4 months’ corrected age, and between IGF-1 concentrations at 4 months and fat and fat-free mass in male infants [49]. These results emphasize the functional relevance of IGF system components in breast milk, especially IGF-1, in influencing both short- and long-term growth outcomes in preterm infants. The higher IGF-1 concentrations observed in preterm milk may reflect an adaptive mechanism to support the increased growth and metabolic demands of premature infants.

### Brain-Derived Neurotrophic Factor

BDNF affects neuronal development, differentiation, and survival, strengthens synaptic connections between nerve cells, and regulates both metabolism and immune function [66, 67]. In colostrum, BDNF levels were markedly reduced in breast milk produced by mothers of preterm infants compared with breast milk produced by mothers of term infants the preterm group compared to the term group [50]. The lower BDNF concentrations observed in preterm colostrum might partially explain the increased neurodevelopmental risk in these infants and suggest a need to explore supportive nutritional interventions.

### Vascular Endothelial Growth Factor

VEGF contributes to angiogenesis and vasculogenesis, promoting the vascular system’s development [68]. As the duration of lactation increases, VEGF levels decrease in breast milk produced by mothers of both preterm and term infants [46, 47]. In one study comparing preterm and term groups, VEGF levels in breast milk were higher in milk produced by mothers of preterm infants at 3, 7, and 28 days of lactation [46]. In another study, the VEGF concentration in breast milk on day 3 of lactation was lower in breast milk produced by mothers of preterm infants, whereas no significant difference was observed between the groups on day 28 of lactation [47]. The discrepancy in VEGF levels between studies may stem from methodological differences, including storage temperatures (− 80 °C vs. −40 °C) and assay techniques (RIA vs. ELISA), which influence protein integrity and detection sensitivity. Such variability underscores the importance of adopting standardized protocols in future studies to enable robust and comparable measurements of bioactive components in breast milk.

### Transforming Growth Factor-α andβ

TGF-α contributes to the repair of the intestinal mucosa and regulates the balance between proliferation and differentiation within the intestinal epithelium [69]. In both the extremely preterm and preterm groups, TGF-α levels in breast milk decreased with the increasing duration of lactation. Furthermore, TGF-α concentrations are higher in breast milk produced by mothers of extremely preterm infants compared to both breast milk produced by mothers of preterm and full-term infants [42].

TGF-β, which is classified as a cytokine by some sources and as a growth factor by others, plays a role in promoting intestinal maturation and defense, supporting the development of the gastrointestinal system, and regulating the immune system [70, 71]. TGF-β has three forms; the most abundant form is TGF-β2 [45, 71]. Colostrum produced by mothers of extremely preterm and preterm infants contained significantly higher levels of TGF-β2 compared with colostrum produced by mothers of term infants [32, 33]. TGF-β2 progressively decreased after delivery, with its highest concentration found in colostrum. Additionally, an inverse relationship was observed between colostrum TGF-β2 levels and both gestational age and birth weight [45]. These findings suggest a compensatory mechanism in extremely preterm milk, with increased concentrations of TGF-α and TGF-β2 possibly supporting the immature gastrointestinal and immune systems of these infants.

### Hepatocyte Growth Factor (HGF)

HGF promotes organogenesis, proliferation, angiogenesis, development of intestine [2, 72]. In a study, the concentration of HGF in colostrum, transitional milk, and mature milk was found to be significantly higher in breast milk produced by mothers of preterm infants compared with that produced by mothers of term infants [52]. Furthermore, HGF levels in breast milk decreased as lactation progressed in both term and preterm groups, but remained at higher levels for a prolonged period in the preterm group [52]. The prolonged elevation of HGF levels in preterm milk could be an adaptive response to accelerate intestinal development and angiogenesis in preterm neonates, helping to bridge the developmental gap caused by early birth.

### Other Growth Factors

In our search, we found a study that investigated the presence of fibroblast growth factor (FGF) and platelet-derived growth factor (PDGF) in breast milk produced by mothers of preterm infants. FGF levels in breast milk produced by mothers of preterm infants were significantly higher than those in breast milk produced by mothers of term infants, whereas PDGF levels were lower, although this difference was not statistically significant [46]. Throughout the breastfeeding period, the FGF levels in the breast milk produced by mothers of preterm infants did not change, whereas the PDGF levels varied significantly [46]. These findings may reflect differential regulatory needs for tissue growth and vascular development in preterm infants, with elevated FGF supporting organogenesis, while fluctuating PDGF levels may indicate a dynamic adaptation to ongoing developmental changes.

## Conclusion

Preterm delivery results in distinct alterations in the concentrations of adipokines and growth factors in breast milk compared to term delivery. In this review, we systematically examined these bioactive components specifically in the breast milk of mothers with preterm infants. While findings regarding leptin and ghrelin remain inconsistent, adiponectin, resistin, and visfatin levels were generally elevated, whereas obestatin levels tended to be lower. In terms of growth factors, preterm delivery was associated with increased levels of EGF, TGF, HGF, and FGF, decreased BDNF, unchanged PDGF levels, and conflicting findings for IGF and VEGF.

These inconsistencies likely stem from heterogeneity in study designs, small sample sizes, variable sampling times across lactation stages, and differences in analytical methodologies. Moreover, interpretation of the findings is further complicated by maternal and environmental factors—including maternal age, BMI, genetic predisposition, nutritional status, environmental exposures, parity, and lactation stage—which independently influence breast milk composition.

Collectively, this review highlights the dynamic, adaptive nature of breast milk in mothers of preterm infants and underscores the potential regulatory roles of specific adipokines and growth factors in shaping neonatal growth, immune system maturation, and neurodevelopment. However, the long-term clinical implications of these components for preterm infant outcomes remain insufficiently elucidated.

Future research should prioritize well-designed, prospective longitudinal studies with larger and more diverse populations, employing standardized protocols for sampling and analysis to enhance comparability across studies. Mechanistic studies aimed at clarifying how these bioactive molecules interact with neonatal metabolic pathways, immune regulation, and organ maturation are also crucial.

Ultimately, advancing our understanding of the functional roles of adipokines and growth factors in breast milk produced by mothers of preterm infants may pave the way for targeted nutritional strategies and personalized interventions aimed at optimizing growth, developmental trajectories, and long-term health in preterm infants.

## Key References

- Qureshi R, Fewtrell M, Wells JCK, Dib S. The association between maternal factors and milk hormone concentrations: a systematic review. Front Nutr. 2024;11:1390232. 10.3389/fnut.2024.1390232.
  - ○ This systematic review provides a comprehensive synthesis of the association between maternal characteristics and breast milk hormone concentrations, offering valuable context for our focus on adipokines and growth factors.
- Szyller H, Antosz K, Batko J, Mytych A, Dziedziak M, Wrześniewska M, et al. Bioactive Components of Human Milk and Their Impact on Child’s Health and Development, Literature Review. Nutrients. 2024;16(10).10.3390/nu16101487.
  - ○ By presenting a comprehensive synthesis of immunological factors, hormones, and growth factors in breast milk, this review offers an essential framework for interpreting the role of adipokines and growth factors in infant outcomes.
- Ramiro-Cortijo D, Singh P, Herranz Carrillo G, Gila-Díaz A, Martín-Cabrejas MA, Martin CR, et al. Association of maternal body composition and diet on breast milk hormones and neonatal growth during the first month of lactation. Front Endocrinol (Lausanne). 2023;14:1090499. 10.3389/fendo.2023.1090499.
  - ○ By examining how maternal body composition and diet influence breast milk hormones and neonatal growth, this study offers a complementary example that enhances the contextualization of our analysis on adipokines in preterm human milk.

## Data Availability

No datasets were generated or analyzed during the current study.

## References

[1] Savino F, Liguori SA, Lupica MM. Adipokines in breast milk and preterm infants. Early Hum Dev. 2010;86(Suppl 1):77–80. 10.1016/j.earlhumdev.2010.01.011. 20129743 10.1016/j.earlhumdev.2010.01.011

[2] Gila-Diaz A, Arribas SM, Algara A, Martín-Cabrejas MA, López de Pablo ÁL, Sáenz de Pipaón M, et al. A review of bioactive factors in human breastmilk: a focus on prematurity. Nutrients. 2019. 10.3390/nu11061307. 31185620 10.3390/nu11061307 PMC6628333

[3] Savino F, Liguori SA, Fissore MF, Oggero R. Breast milk hormones and their protective effect on obesity. Int J Pediatr Endocrinol. 2009;2009:327505. 10.1155/2009/327505. 20049153 10.1155/2009/327505 PMC2798107

[4] Fields DA, Demerath EW. Relationship of insulin, glucose, leptin, IL-6 and TNF-α in human breast milk with infant growth and body composition. Pediatr Obes. 2012;7(4):304–12. 10.1111/j.2047-6310.2012.00059.x. 22577092 10.1111/j.2047-6310.2012.00059.x PMC3393795

[5] Ballard O, Morrow AL. Human milk composition: nutrients and bioactive factors. Pediatr Clin North Am. 2013;60(1):49–74. 10.1016/j.pcl.2012.10.002. 23178060 10.1016/j.pcl.2012.10.002 PMC3586783

[6] Lugonja N, Marinković V, Pucarević M, Miletić S, Stojić N, Crnković D, et al. Human milk-the biofluid that nourishes infants from the first day of life. Foods. 2024. 10.3390/foods13091298. 38731669 10.3390/foods13091298 PMC11083309

[7] Gidrewicz DA, Fenton TR. A systematic review and meta-analysis of the nutrient content of preterm and term breast milk. BMC Pediatr. 2014;14:216. 10.1186/1471-2431-14-216. 25174435 10.1186/1471-2431-14-216 PMC4236651

[8] Bauer J, Gerss J. Longitudinal analysis of macronutrients and minerals in human milk produced by mothers of preterm infants. Clin Nutr. 2011;30(2):215–20. 10.1016/j.clnu.2010.08.003. 20801561 10.1016/j.clnu.2010.08.003

[9] Fischer Fumeaux CJ, Garcia-Rodenas CL, De Castro CA, Courtet-Compondu MC, Thakkar SK, Beauport L, et al. Longitudinal analysis of macronutrient composition in preterm and term human milk: a prospective cohort study. Nutrients. 2019. 10.3390/nu11071525. 31277502 10.3390/nu11071525 PMC6683284

[10] Erliana UD, Fly AD. The function and alteration of immunological properties in human milk of obese mothers. Nutrients. 2019. 10.3390/nu11061284. 31174304 10.3390/nu11061284 PMC6627488

[11] Garofoli F, Civardi E, Pisoni C, Angelini M, Ghirardello S. Anti-inflammatory and anti-allergic properties of colostrum from mothers of full-term and preterm babies: the importance of maternal lactation in the first days. Nutrients. 2023. 10.3390/nu15194249. 37836533 10.3390/nu15194249 PMC10574092

[12] Andreas NJ, Kampmann B, Mehring Le-Doare K. Human breast milk: a review on its composition and bioactivity. Early Hum Dev. 2015;91(11):629–35. 10.1016/j.earlhumdev.2015.08.013. 26375355 10.1016/j.earlhumdev.2015.08.013

[13] Andreas NJ, Hyde MJ, Gale C, Parkinson JR, Jeffries S, Holmes E, et al. Effect of maternal body mass index on hormones in breast milk: a systematic review. PLoS One. 2014;9(12):e115043. 10.1371/journal.pone.0115043. 25536196 10.1371/journal.pone.0115043 PMC4275218

[14] Young BE, Johnson SL, Krebs NF. Biological determinants linking infant weight gain and child obesity: current knowledge and future directions. Adv Nutr. 2012;3(5):675–86. 10.3945/an.112.002238. 22983846 10.3945/an.112.002238 PMC3648749

[15] Fields DA, Schneider CR, Pavela G. A narrative review of the associations between six bioactive components in breast milk and infant adiposity. Obes (Silver Spring). 2016;24(6):1213–21. 10.1002/oby.21519. 10.1002/oby.21519 PMC5325144 27151491

[16] Ohuma EO, Moller AB, Bradley E, Chakwera S, Hussain-Alkhateeb L, Lewin A, et al. National, regional, and global estimates of preterm birth in 2020, with trends from 2010: a systematic analysis. Lancet. 2023;402(10409):1261–71. 10.1016/s0140-6736(23)00878-4. 37805217 10.1016/S0140-6736(23)00878-4

[17] Lawn JE, Gravett MG, Nunes TM, Rubens CE, Stanton C. Global report on preterm birth and stillbirth (1 of 7): definitions, description of the burden and opportunities to improve data. BMC Pregnancy Childbirth. 2010;10(1):S1. 10.1186/1471-2393-10-s1-s1. 20233382 10.1186/1471-2393-10-S1-S1 PMC2841772

[18] Victora CG, Bahl R, Barros AJ, França GV, Horton S, Krasevec J, et al. Breastfeeding in the 21st century: epidemiology, mechanisms, and lifelong effect. Lancet. 2016;387(10017):475–90. 10.1016/s0140-6736(15)01024-7. 26869575 10.1016/S0140-6736(15)01024-7

[19] Contreras Chova F, Villanueva-García A, González-Boyero JL, Campos-Martínez AM, Blanca-Jover E, Jerez-Calero AE, et al. Strategies for the fortification of human milk in preterm infants: a systematic review. Cureus. 2024;16(11):e73380. 10.7759/cureus.73380. 39659332 10.7759/cureus.73380 PMC11630059

[20] Shelby RD, Cromeens B, Rager TM, Besner GE. Influence of growth factors on the development of necrotizing enterocolitis. Clin Perinatol. 2019;46(1):51–64. 10.1016/j.clp.2018.10.005. 30771819 10.1016/j.clp.2018.10.005 PMC6380490

[21] Weyermann M, Brenner H, Rothenbacher D. Adipokines in human milk and risk of overweight in early childhood: a prospective cohort study. Epidemiology. 2007;18(6):722–9. 10.1097/ede.0b013e3181567ed4. 18062063 10.1097/ede.0b013e3181567ed4

[22] Aryadevi NNB, Putri RSI, Marsubrin PMT. Human breast milk fortification with human milk fortifier vs preterm infant formula: a systematic review and meta-analysis. J Neonatal Perinatal Med. 2025;19345798251350987. 10.1177/19345798251350987. 10.1177/19345798251350987 40534564

[23] Arslanoglu S, Boquien CY, King C, Lamireau D, Tonetto P, Barnett D, et al. Fortification of human milk for preterm infants: update and recommendations of the European Milk Bank Association (EMBA) working group on human milk fortification. Front Pediatr. 2019;7:76. 10.3389/fped.2019.00076. 30968003 10.3389/fped.2019.00076 PMC6439523

[24] Zivaljevic J, Jovandaric MZ, Babic S, Raus M. Complications of preterm birth-the importance of care for the outcome: a narrative review. Medicina (Kaunas). 2024. 10.3390/medicina60061014. 38929631 10.3390/medicina60061014 PMC11205595

[25] Clark RH, Thomas P, Peabody J. Extrauterine growth restriction remains a serious problem in prematurely born neonates. Pediatrics. 2003;111(5 Pt 1):986–90. 10.1542/peds.111.5.986. 12728076 10.1542/peds.111.5.986

[26] Lawrence RM, Pane CA. Human breast milk: current concepts of immunology and infectious diseases. Curr Probl Pediatr Adolesc Health Care. 2007;37(1):7–36. 10.1016/j.cppeds.2006.10.002. 17157245 10.1016/j.cppeds.2006.10.002

[27] Collado MC, Santaella M, Mira-Pascual L, Martínez-Arias E, Khodayar-Pardo P, Ros G, et al. Longitudinal study of cytokine expression, lipid profile and neuronal growth factors in human breast milk from term and preterm deliveries. Nutrients. 2015;7(10):8577–91. 10.3390/nu7105415. 26492267 10.3390/nu7105415 PMC4632435

[28] Çatlı G, Olgaç Dündar N, Dündar BN. Adipokines in breast milk: an update. J Clin Res Pediatr Endocrinol. 2014;6(4):192–201. 10.4274/Jcrpe.1531. 25541889 10.4274/jcrpe.1531 PMC4293653

[29] Balcells-Esponera C, Borràs-Novell C, López-Abad M, Cubells Serra I, Basseda Puig A, Izquierdo Renau M, et al. Bioactive peptides in preterm human milk: Impact of maternal characteristics and their association to neonatal outcomes. Biofactors. 2024;50(1):135–44. 10.1002/biof.1997. 37584623 10.1002/biof.1997

[30] Castellote C, Casillas R, Ramírez-Santana C, Pérez-Cano FJ, Castell M, Moretones MG, et al. Premature delivery influences the immunological composition of colostrum and transitional and mature human milk. J Nutr. 2011;141(6):1181–7. 10.3945/jn.110.133652. 21508211 10.3945/jn.110.133652

[31] Mehta R, Petrova A. Biologically active breast milk proteins in association with very preterm delivery and stage of lactation. J Perinatol. 2011;31(1):58–62. 10.1038/jp.2010.68. 20523299 10.1038/jp.2010.68

[32] Kociszewska-Najman B, Sibanda E, Radomska-Leśniewska DM, Taradaj K, Kociołek P, Ginda T, et al. Does caesarean section or preterm delivery influence TGF-β2 concentrations in human colostrum? Nutrients. 2020. 10.3390/nu12041095. 32326558 10.3390/nu12041095 PMC7230194

[33] Trend S, Strunk T, Lloyd ML, Kok CH, Metcalfe J, Geddes DT, et al. Levels of innate immune factors in preterm and term mothers’ breast milk during the 1st month postpartum. Br J Nutr. 2016;115(7):1178–93. 10.1017/s0007114516000234. 26891901 10.1017/S0007114516000234

[34] Caba-Flores MD, Cardenas-Tueme M, Viveros-Contreras R, Martínez-Valenzuela C, Zurutuza-Lorméndez JI, Ortiz-López R, et al. Preterm Delivery in Obese Mothers Predicts Tumor Necrosis Factor-α Levels in Breast Milk. Breastfeed Med. 2023;18(12):934–42. 10.1089/bfm.2023.0153. 38100442 10.1089/bfm.2023.0153

[35] Ramiro-Cortijo D, Singh P, Herranz Carrillo G, Gila-Díaz A, Martín-Cabrejas MA, Martin CR, et al. Association of maternal body composition and diet on breast milk hormones and neonatal growth during the first month of lactation. Front Endocrinol (Lausanne). 2023;14:1090499. 10.3389/fendo.2023.1090499. 36936154 10.3389/fendo.2023.1090499 PMC10018215

[36] Kocaadam B, Koksal E, Ozcan KE, Turkyilmaz C. Do the adiponectin and leptin levels in preterm and term breast milk samples relate to infants’ short-term growth? J Dev Orig Health Dis. 2019;10(2):253–8. 10.1017/s2040174418000703. 30203736 10.1017/S2040174418000703

[37] Slupecka-Ziemilska M, Wolinski J, Herman AP, Romanowicz K, Dziegielewska Z, Borszewska-Kornacka MK. Influence of preterm delivery on ghrelin and obestatin concentrations in maternal plasm, milk and their expression in mammary epithelial cells. J Physiol Pharmacol. 2017;68(5):693–8. 29375043

[38] Eilers E, Ziska T, Harder T, Plagemann A, Obladen M, Loui A. Leptin determination in colostrum and early human milk from mothers of preterm and term infants. Early Hum Dev. 2011;87(6):415–9. 10.1016/j.earlhumdev.2011.03.004. 21482454 10.1016/j.earlhumdev.2011.03.004

[39] Oslislo A, Czuba Z, Sławska H, Kaźmierczak W, Król W. Decreased human milk concentration of epidermal growth factor after preterm delivery of intrauterine growth-restricted newborns. J Pediatr Gastroenterol Nutr. 2007;44(4):464–7. 10.1097/MPG.0b013e3180331e15. 17414145 10.1097/MPG.0b013e3180331e15

[40] Kierson JA, Dimatteo DM, Locke RG, Mackley AB, Spear ML. Ghrelin and cholecystokinin in term and preterm human breast milk. Acta Paediatr. 2006;95(8):991–5. 10.1080/08035250600669769. 16882575 10.1080/08035250600669769

[41] Bielicki J, Huch R, von Mandach U. Time-course of leptin levels in term and preterm human milk. Eur J Endocrinol. 2004;151(2):271–6. 10.1530/eje.0.1510271. 15296484 10.1530/eje.0.1510271

[42] Dvorak B, Fituch CC, Williams CS, Hurst NM, Schanler RJ. Increased epidermal growth factor levels in human milk of mothers with extremely premature infants. Pediatr Res. 2003;54(1):15–9. 10.1203/01.Pdr.0000065729.74325.71. 12646719 10.1203/01.PDR.0000065729.74325.71

[43] Mól N, Zasada M, Tomasik P, Klimasz K, Kwinta P. Evaluation of irisin and visfatin levels in very low birth weight preterm newborns compared to full term newborns-a prospective cohort study. PLoS One. 2018;13(9):e0204835. 10.1371/journal.pone.0204835. 30261060 10.1371/journal.pone.0204835 PMC6160155

[44] Groer M, Ashmeade T, Duffy A, Morse S, Zaritt J. Changes in the immune components of preterm human milk and associations with maternal and infant characteristics. J Obstet Gynecol Neonatal Nurs. 2016;45(5):639–48. 10.1016/j.jogn.2016.04.009. 27477269 10.1016/j.jogn.2016.04.009

[45] Frost BL, Jilling T, Lapin B, Maheshwari A, Caplan MS. Maternal breast milk transforming growth factor-beta and feeding intolerance in preterm infants. Pediatr Res. 2014;76(4):386–93. 10.1038/pr.2014.96. 24995914 10.1038/pr.2014.96 PMC4467901

[46] Ozgurtas T, Aydin I, Turan O, Koc E, Hirfanoglu IM, Acikel CH, et al. Vascular endothelial growth factor, basic fibroblast growth factor, insulin-like growth factor-I and platelet-derived growth factor levels in human milk of mothers with term and preterm neonates. Cytokine. 2010;50(2):192–4. 10.1016/j.cyto.2010.02.008. 20202860 10.1016/j.cyto.2010.02.008

[47] Loui A, Eilers E, Strauss E, Pohl-Schickinger A, Obladen M, Koehne P. Vascular endothelial growth factor (VEGF) and soluble VEGF receptor 1 (sFlt-1) levels in early and mature human milk from mothers of preterm versus term infants. J Hum Lact. 2012;28(4):522–8. 10.1177/0890334412447686. 22729710 10.1177/0890334412447686

[48] Binder C, Baumgartner-Parzer S, Gard LI, Berger A, Thajer A. Maternal diet influences human milk protein concentration and adipose tissue marker. Nutrients. 2023. 10.3390/nu15020433. 36678304 10.3390/nu15020433 PMC9866185

[49] Galante L, Reynolds CM, Milan AM, Alexander T, Bloomfield FH, Cameron-Smith D, et al. Preterm human milk: associations between perinatal factors and hormone concentrations throughout lactation. Pediatr Res. 2021;89(6):1461–9. 10.1038/s41390-020-1069-1. 32726796 10.1038/s41390-020-1069-1

[50] Nyárády K, Turai R, Funke S, Györgyi E, Makai A, Prémusz V, et al. Effects of perinatal factors on sirtuin 3, 8-hydroxy-2’- deoxyguanosine, brain-derived neurotrophic factor and serotonin in cord blood and early breast milk: an observational study. Int Breastfeed J. 2020;15(1):57. 10.1186/s13006-020-00301-z. 32552911 10.1186/s13006-020-00301-z PMC7302386

[51] Elmlinger MW, Hochhaus F, Loui A, Frommer KW, Obladen M, Ranke MB. Insulin-like growth factors and binding proteins in early milk from mothers of preterm and term infants. Horm Res. 2007;68(3):124–31. 10.1159/000100488. 17341887 10.1159/000100488

[52] Itoh H, Itakura A, Kurauchi O, Okamura M, Nakamura H, Mizutani S. Hepatocyte growth factor in human breast milk acts as a trophic factor. Horm Metab Res. 2002;34(1):16–20. 10.1055/s-2002-19961. 11832996 10.1055/s-2002-19961

[53] Joung KE, Martin CR, Cherkerzian S, Kellogg M, Belfort MB. Human Milk Hormone Intake in the First Month of Life and Physical Growth Outcomes in Preterm Infants. J Clin Endocrinol Metab. 2021;106(6):1793–803. 10.1210/clinem/dgab001. 33544860 10.1210/clinem/dgab001

[54] Qureshi R, Fewtrell M, Wells JCK, Dib S. The association between maternal factors and milk hormone concentrations: a systematic review. Front Nutr. 2024;11:1390232. 10.3389/fnut.2024.1390232. 39021603 10.3389/fnut.2024.1390232 PMC11253774

[55] Palou M, Picó C, Palou A. Leptin as a breast milk component for the prevention of obesity. Nutr Rev. 2018;76(12):875–92. 10.1093/nutrit/nuy046. 30285146 10.1093/nutrit/nuy046

[56] Suwaydi MA, Gridneva Z, Perrella SL, Wlodek ME, Lai CT, Geddes DT. Human milk metabolic hormones: analytical methods and current understanding. Int J Mol Sci. 2021. 10.3390/ijms22168708. 34445437 10.3390/ijms22168708 PMC8395916

[57] Badillo-Suárez PA, Rodríguez-Cruz M, Nieves-Morales X. Impact of metabolic hormones secreted in human breast milk on nutritional programming in childhood obesity. J Mammary Gland Biol Neoplasia. 2017;22(3):171–91. 10.1007/s10911-017-9382-y. 28653126 10.1007/s10911-017-9382-y

[58] Aydin S, Ozkan Y, Erman F, Gurates B, Kilic N, Colak R, et al. Presence of obestatin in breast milk: relationship among obestatin, ghrelin, and leptin in lactating women. Nutrition. 2008;24(7–8):689–93. 10.1016/j.nut.2008.03.020. 18499397 10.1016/j.nut.2008.03.020

[59] Sethi JK, Vidal-Puig A. Visfatin: the missing link between intra-abdominal obesity and diabetes? Trends Mol Med. 2005;11(8):344–7. 10.1016/j.molmed.2005.06.010. 16005682 10.1016/j.molmed.2005.06.010 PMC4303999

[60] Warner BW, Warner BB. Role of epidermal growth factor in the pathogenesis of neonatal necrotizing enterocolitis. Semin Pediatr Surg. 2005;14(3):175–80. 10.1053/j.sempedsurg.2005.05.006. 16084405 10.1053/j.sempedsurg.2005.05.006

[61] Patel L, Cavazzoni E, Whatmore AJ, Carney S, Wales JK, Clayton PE, et al. The contributions of plasma IGF-I, IGFBP-3 and leptin to growth in extremely premature infants during the first two years. Pediatr Res. 2007;61(1):99–104. 10.1203/01.pdr.0000250036.34522.f1. 17211149 10.1203/01.pdr.0000250036.34522.f1

[62] Szyller H, Antosz K, Batko J, Mytych A, Dziedziak M, Wrześniewska M, et al. Bioactive components of human milk and their impact on child’s health and development, literature review. Nutrients. 2024. 10.3390/nu16101487. 38794725 10.3390/nu16101487 PMC11124180

[63] Tang X, Liu H, Yang S, Li Z, Zhong J, Fang R. Epidermal growth factor and intestinal barrier function. Mediators Inflamm. 2016;2016:1927348. 10.1155/2016/1927348. 27524860 10.1155/2016/1927348 PMC4976184

[64] Hoeflich A, Meyer Z. Functional analysis of the IGF-system in milk. Best Pract Res Clin Endocrinol Metab. 2017;31(4):409–18. 10.1016/j.beem.2017.10.002. 29221569 10.1016/j.beem.2017.10.002

[65] York DJ, Smazal AL, Robinson DT, De Plaen IG. Human milk growth factors and their role in NEC prevention: a narrative review. Nutrients. 2021. 10.3390/nu13113751. 34836007 10.3390/nu13113751 PMC8620589

[66] Numakawa T, Suzuki S, Kumamaru E, Adachi N, Richards M, Kunugi H. Bdnf function and intracellular signaling in neurons. Histol Histopathol. 2010;25(2):237–58. 10.14670/hh-25.237. 20017110 10.14670/HH-25.237

[67] Johne M, Maculewicz E, Mastalerz A, Białek M, Wojtak W, Osuch B, et al. Dietary patterns, serum BDNF and fatty acid profiles in physically active male young adults: a cluster analysis study. Nutrients. 2024. 10.3390/nu16244326. 39770947 10.3390/nu16244326 PMC11679842

[68] Froń A, Orczyk-Pawiłowicz M. Understanding the immunological quality of breast milk in maternal overweight and obesity. Nutrients. 2023. 10.3390/nu15245016. 38140275 10.3390/nu15245016 PMC10746120

[69] Playford RJ, Macdonald CE, Johnson WS. Colostrum and milk-derived peptide growth factors for the treatment of gastrointestinal disorders. Am J Clin Nutr. 2000;72(1):5–14. 10.1093/ajcn/72.1.5. 10871554 10.1093/ajcn/72.1.5

[70] Lewis ED, Richard C, Larsen BM, Field CJ. The importance of human milk for immunity in preterm infants. Clin Perinatol. 2017;44(1):23–47. 10.1016/j.clp.2016.11.008. 28159208 10.1016/j.clp.2016.11.008

[71] Thai JD, Gregory KE. Bioactive factors in human breast milk attenuate intestinal inflammation during early life. Nutrients. 2020. 10.3390/nu12020581. 32102231 10.3390/nu12020581 PMC7071406

[72] Kobata R, Tsukahara H, Ohshima Y, Ohta N, Tokuriki S, Tamura S, et al. High levels of growth factors in human breast milk. Early Hum Dev. 2008;84(1):67–9. 10.1016/j.earlhumdev.2007.07.005. 17716837 10.1016/j.earlhumdev.2007.07.005

